# Inherent Lipid Composition Abnormalities in Astrocytes Associated with Late-Onset Alzheimer’s Disease (LOAD)

**DOI:** 10.3390/cells15060549

**Published:** 2026-03-19

**Authors:** Bruce M. Cohen, Eunjung Koh, Kandice R. Levental, Ilya Levental, Kai-Christian Sonntag

**Affiliations:** 1 Program for Neuropsychiatric Research, McLean Hospital, 115 Mill St., Belmont, MA 02478, USA; ekoh@mclean.harvard.edu (E.K.); kai@mclean.harvard.edu (K.-C.S.); 2 Laboratory for Translational Research on Neurodegeneration, McLean Hospital, 115 Mill St., Belmont, MA 02478, USA; 3 Department of Molecular Physiology and Biological Physics, University of Virginia School of Medicine, 200 Jeanette Lancaster Way, Charlottesville, VA 22903, USA; krl6c@virginia.edu (K.R.L.); il2sy@virginia.edu (I.L.)

**Keywords:** late-onset Alzheimer’s disease, LOAD, lipids, induced pluripotent stem cells, iPSC, astrocytes, mitochondria, cholesterol, fatty acids, dementia risk

## Abstract

**Highlights:**

**What are the main findings?**

- There are substantial reductions in cholesterol esters in astrocytes derived from cells of patients with late-onset Alzheimer’s disease (LOAD).
- Most fatty acids in membrane lipids of these astrocytes, including in mitochondrial membranes, tend to be reduced, with only a few increased.

**What are the implications of the main findings?**

- Because these astrocytes are derived from induced pluripotent stem cell (iPSC) lines, the abnormalities observed are inherent features of the cells and may be the determinants of LOAD.
- These abnormalities may be targets for interventions that would reduce the risk for LOAD or slow its progression.

**Abstract:**

Lipid abnormalities have been observed in brain, cerebrospinal fluid (CSF), and blood in association with late-onset Alzheimer’s disease (LOAD). It is unknown which of these abnormalities are precursors to LOAD and which are concomitants of illness or its treatment. Inherent abnormalities can be identified in induced pluripotent stem cell (iPSC)-derived brain cells. These cells lack markers associated with aging and environmental exposures. The iPSC lines of patients with LOAD or healthy individuals were differentiated to astrocytes. Astrocytes are crucial to neural activity and health, and altered astrocyte functions are associated with LOAD pathology. Lipidomics analyses were performed on whole-cell and mitochondria-enriched fractions. Large reductions in cholesterol esters (CEs) and imbalances in fatty acids (FAs) were observed in LOAD-associated cells or their mitochondria. There were only modest differences in other lipid classes, including membrane structural lipids. The findings identify abnormalities in CEs, as well as in FAs, as inherent abnormalities and likely precursors to LOAD. These differences implicate mechanisms contributing to disease pathogenesis. Further study may lead to early interventions to prevent or delay LOAD.

## 1. Introduction

Over 90% of dementias present after the age of 65, and in most of these cases, LOAD is diagnosed [1].

Unlike early-onset Alzheimer’s disease (EOAD), which is usually the consequence of highly penetrant variants in single genes, the inherent risk for LOAD is determined by interactions among numerous genes, possibly hundreds of them, with low penetrance [2]. These genes underlie multiple processes needed for cell development, repair and remodeling, and responses to aging and environmental exposures. Through effects on all these functions, they determine the inherent risk for LOAD. These inherent risk factors interact, through time, with environmental factors leading to the development of illness in those with higher risk factors. The studies presented here examine one of these factors: inherent abnormalities of lipids in cells from people with LOAD. As background supporting the role of lipids and to put the results in context, evidence suggesting lipid abnormalities as determinants of risk for LOAD is reviewed.

### 1.1. Genetic Factors Linking Lipids with LOAD

Both single gene findings and pathway analyses suggest that differences in lipid metabolism strongly affect risk [2,3,4]. This includes gene sets associated with cholesterol synthesis, transport, and metabolism, as well as gene sets associated with FAs and phospholipid synthesis and metabolism [5,6].

The strongest gene association for LOAD is with the E4 variant of the apolipoprotein ε (*APOE*) gene, which codes for a lipoprotein that transports lipids, predominantly cholesterol and cholesterol esters, as well as FAs, between and within cells [7]. About 25% of people are heterozygous, while 2–3% are homozygous for *APOE4*, and 40–65% of patients with AD carry the *APOE4* gene. A single copy of E4 increases risk for AD 3- to 7-fold, and homozygosity increases risk 12- to 15-fold [8]. While the E3 variant is the most common, the E2 variant is associated with a decreased risk for LOAD. APOE4 may increase the risk for LOAD both through its effects on lipid transport in brain and through its effects on lipid accumulations on the walls of blood vessels supplying the brain [9,10].

### 1.2. Epidemiological Factors Suggesting an Association of Lipids with LOAD

Diets rich in saturated fats can increase the risk for LOAD [11]. And high levels of some fats and cholesterol in blood, packaged with APOE in low-density lipoproteins (LDL), are associated with an increased risk for dementia [12]. Interventions that reduce cholesterol levels, including treatment with statins, reduce that risk [13].

However, if there is an intact blood–brain barrier, plasma cholesterol does not freely enter the brain [14,15]. Rather, cholesterol is made in the brain, and there is evidence, from studies of cholesterol and its precursors in CSF, that patients with LOAD have decreased, not increased, production of cholesterol in brain [15,16]. The association of blood and dietary cholesterol with poorer brain health may largely be a consequence of the effects of blood cholesterol on vascular health [17,18]. Specifically, atherosclerosis is associated with decreased blood flow to brain, thereby compromising brain function and repair. In addition, damage to vasculature integrity, especially to the blood–brain barrier, exposes the brain to toxins in the circulation.

The role of FAs is more debated than the role of cholesterol, but elevated saturated fats in diet and blood may be associated with an increased risk for dementia. In this case, as in the case of cholesterol, the causal mechanisms may be indirect. Saturated FAs, versus unsaturated or polyunsaturated FAs, induce inflammatory activity with widespread toxicity, including damage to the cardiovascular system and, through blood-borne inflammatory molecules, in the brain [19].

### 1.3. The Role of Lipids and Their Relationships to the Risk of Illness

It is easy to understand why lipid types and levels in brain might directly contribute to risk for LOAD. Most cellular processes occur across, along, or within membranous elements and organelles of cells [20]. Lipids are key components of all cell membranes, and lipid content abnormalities can alter many physiologic functions known to be associated with LOAD. These include glucose uptake and glycolysis, protein processing in the Golgi apparatus and endoplasmic reticulum (ER), energy production in mitochondria, the digestion and elimination of toxic compounds in lysosomes and autophagosomes, the storage and release of metabolic precursors, and the storage and release of signaling molecules, which include Ca^2+^, cholesterol esters, and FAs that modulate cell functions [20,21,22]. Also, the aggregation of toxic A-beta (Aβ) amyloid fragments, underlying the amyloid plaques often seen in LOAD, has been linked to Aβ interactions with specific lipids in cell membranes [13].

### 1.4. Background on Determining Inherent Lipid Abnormalities Associated with LOAD

In this paper, we briefly review reports on lipid content observed in postmortem brain tissue, CSF, and plasma in people with LOAD. Some studies included people with mild cognitive impairment (MCI), which often precedes LOAD. Because the measures in these studies were determined in samples from people who were symptomatic, it is not known with certainty which of the abnormalities observed are basic factors underlying risk for LOAD, and which are a consequence of illness and its treatment [23]. Some findings observed when illness is established may be direct consequences of inherent factors, but they may also reflect physiologic compensations, treatment effects, other environmental and experiential factors, and in the case of LOAD, aging. It is important to identify inherent abnormalities because they may be addressable before consequential illness, along with irreversible tissue damage. 

To address this question, we studied lipids in reprogrammed cells obtained from individuals with LOAD. Fibroblasts or blood cells were converted to iPSC, differentiated into neural progenitor cells (NPCs), and then into more mature brain cells. Such reprogrammed cells, produced from iPSC lines, lack most or all epigenetic or other features associated with aging, illness, and its treatment. Lipid abnormalities in these cells are, therefore, inherent to the lines. They are likely to be abnormalities that underly neuropathological processes rather than being consequences of contributing to the development of LOAD [22].

### 1.5. Past Studies of Lipid Classes in Individuals with LOAD

Most lipids analyzed in people with LOAD fall into the following classes: (1) unesterified cholesterol and cholesterol esters, (2) glycerophospholipids and their derivatives, and (3) sphingolipids, including ceramides and sphingomyelin [20]. Cholesterol, glycerophospholipids, and sphingolipids are present at substantial concentrations in all cell membranes, but their relative proportions differ among membranes [20,23]. Cholesterol is highest in the external cell membrane, and phosphatidylcholine, one of several common glycerophospholipids, is the highest concentration lipid in most other (internal) cell membranes. Sphingolipids are present at lower concentrations in both external and internal membranes. Lipids in each class can have side chains, most commonly, FAs. These can be exchanged among lipids of different classes. They are also seen free or within lipid droplets in cells.

### 1.6. Cholesterol and Its Derivatives in LOAD

Cholesterol in most tissues, including brain, exists in two pools, neutral cholesterol and cholesterol esterified with FAs (CEs). In membranes, neutral cholesterol is the core element of lipid rafts that house membrane-associated proteins, including receptors, ion channels, and enzyme complexes, all necessary for basic cell function [21]. Elevated cholesterol levels in blood are associated with a higher risk for dementia; cholesterol does not appear to be increased in peripheral or brain cell membranes in people with LOAD [24]. Cholesterol levels in brain increase with age, and while they are not substantially altered in postmortem brain tissue in LOAD, both cholesterol synthesis and degradation may be reduced in LOAD [25]. These changes may be both a consequence and a driver of Aβ formation [23]. That is, Aβ affects membrane composition and membrane composition affects amyloid processing.

CEs are storage and signaling molecules. They can be stored in lipid droplets and can be hydrolyzed back to free cholesterol by several cholesterol esterases [2]. The cholesterol ester cycle is a crucial mechanism of cell functions in most mammalian tissue, with brain tissue having the highest rates of de novo cholesterol biosynthesis [26]. As a messenger, CE can directly influence numerous cell processes, including those related to bioenergetics, protein processing, autophagy, and inflammation [27]. Lipid droplets, which also contain other neutral lipids, have been reported to be elevated in brain in postmortem studies of LOAD [7,28]. CEs were observed to be elevated in the frontal cortex [29] or only in diseased entorhinal cortex and not frontal cortex in association with LOAD [30]. It is not known if these increases are precursors or consequences of neurodegeneration in LOAD [21]. In vivo, CE was observed to be decreased in plasma in people with LOAD [31]. Since that first report, it has consistently been observed that CE is substantially decreased in both plasma and CSF of people with LOAD [32,33]. Low CE provided good classification accuracy for identifying individuals with LOAD [33]. As a possible mechanism for these findings, cholesterol esterification rate was reduced in CSF from patients with LOAD versus healthy controls [34]. And genes associated with CE metabolism are also associated with LOAD [33]. Addressing the stage at which this abnormality occurs, individuals with MCI have levels of CE between those seen in people with LOAD and healthy comparison subjects, and lower levels of CE in MCI have been suggested as a maker for MCI conversion to LOAD [24]. These genomic and mechanistic associations, along with replicated observations of substantially low CEs in vivo in LOAD and in people with MCI who proceed to LOAD, suggest that low CE levels might be a precursor of risk for LOAD.

### 1.7. Glycerophospholipids and Their Derivatives in LOAD

Among glycerophospholipids, phosphatidylcholine (PC) has been the most studied regarding an association with LOAD. This is in part because PC is the most prominent glycerophospholipid and a main component of cholinergic neurons, serving both as a structural lipid and as a precursor for producing the neurotransmitter acetylcholine [35,36]. Dysfunction, shrinkage, and death of long axon cholinergic neurons in the brain are among the earliest and strongest signs of Alzheimer’s disease [37,38]. Brain choline uptake and metabolism decrease with age, and this decline may increase the risk for the degeneration of cholinergic neurons [39,40]. Medications that increase cholinergic signaling were the first drugs developed and marketed for the treatment of LOAD, and they remain in use today [41].

PC has most often been reported to be reduced in postmortem tissue, as well as in CSF and blood, from individuals with LOAD [3,21]. However, some studies observed increased or normal PC in brain samples of people with LOAD [42]. Similar to findings with PC, phosphatidylethanolamine (PE), phosphatidylinositol (PI), and phosphatidylserine (PS), along with phosphatidic acid (PA), have all been reported to be decreased in post-mortem brain tissue from people with LOAD [3,21]. As a whole, the results suggest that most glycerophospholipids are decreased in tissue samples from people diagnosed with LOAD.

Lysoglycerophospholipids have only one FA side chain. They have been less studied than glycerophospholipids, which have two FA side chains. Lysophosphatidyls, including lysophosphatidylcholine (LPC) and lysophosphatidylethanolamine (LPE), are enzymatically broken-down products from PC or PE by removing the FA chain. In the Lands cycle, PC and PE can be reconverted from LPC or LPE through acyltransferases. While high levels of LPC can be lipotoxic, LPE is cell-protective. The levels of these phospholipids and their ratios can reveal information on membrane disruptions and have been implicated as factors and biomarkers in human disease, including AD [43]. In a postmortem study, both PC and LPC were observed to be abnormally high and correlated positively with Aβ content in brains of patients with LOAD or intermediate probability of LOAD, and some PE or LPE species were also elevated [4]. However, another postmortem study revealed lower levels of LPC and LPE and reduced LPC/PC and LPE/PE ratios in brains from asymptomatic, as well as symptomatic, AD patients [44]. In CSF, LPC, individually, and the ratio of LPC/PC were low in people with LOAD [45]. In plasma, one study observed that both LPC and PC, individually, as well as the ratio of LPC/PC, were low in people who concerted to LOAD [46]. Different levels of PE, LPC, and LPE or their ratios have also been observed in other studies on serum or plasma, and it was suggested that these lipids could be biomarkers for MCI and MCI conversion to AD [47,48].

Phosphatidylglycerol (PG), which has a glycerol backbone and a charged head group but no FA side chains, has been observed to be elevated in postmortem studies of MCI and LOAD [4]. The levels of PG were strongly associated with the number of plaques and tangles in brain in this investigation.

Diacylglycerol (DAG), a glycerophospholipid with two FA side chains and no charged head group, was reported to be significantly elevated in plasma and postmortem brain tissue of people with MCI or early LOAD [23,33,49].

### 1.8. Sphingolipids in LOAD

Sphingolipids, like glycerophospholipids, are a class of compounds with varying FA side chains and polar head groups. Examples include sphingomyelin, found in many cell membranes; ceramides, which are part of cell membranes and also act as second messengers; and gangliosides, a large family of glycosphingolipids highly represented in lipid rafts within plasma membranes of cells in the brain, where they modulate the activity of ion channels [50]. Once again illustrating the many roles of lipids, gangliosides also serve as recognition and communication molecules between cells [51].

Studies on postmortem tissue show altered profiles of sphingolipids in LOAD [23]. However, the studies do not consistently agree on which sphingolipids are found at higher and lower levels in postmortem brain tissue [3,50]. This may be because sphingolipid levels in brain change both with age and through the course of illness [42]. The entire class is highly interactive with other factors observed to be abnormal in LOAD, including other lipids [52]. In addition, they are reactive to stress and immune functions [28]. Studies of ceramides suggest that levels may be increased early in illness and decreased at later times [4,28].

### 1.9. Fatty Acids in LOAD

FAs in the brain and periphery exist either as side chains, conjugated to other lipids, or as free FAs. The latter are stored in lipid droplets or are used as signaling molecules within the cell. In most studies of people with LOAD, no analyses to determine the relative amount or proportion of different FA types were performed or reported. When studied, FAs, as a group, appeared to be low in postmortem samples of brain from people with LOAD [4,53], with the possible exception of docosahexaenoic acid (DHA) [53]. In lipid rafts isolated from postmortem brain tissue, polyunsaturated FAs, including DHA, were low in samples from people with LOAD [54]. In a postmortem study of FAs attached to phosphatidylcholines, all classes of FAs were observed to be abnormally low, with unsaturated FAs being particularly low [55]. In addition to steady-state studies of FA levels, there have been studies explicitly implicating associations between FAs, including medium and saturated long-chain fatty acids (VLCFAs) in blood and CSF and progression from MCI to LOAD [56,57,58]. Regarding possible mechanisms underlying abnormalities of FA metabolism, it can be noted that VLCFAs in the brain are broken down in peroxisomes through β-oxidation to maintain FA homeostasis and lipid contents of membranes. In postmortem brain tissue in LOAD, peroxisomes have been observed to rise and fall over time [59], and altered FA levels, including increases in VLCFAs, may be related to concurrent loss or dysfunction of peroxisomes [60].

Aside from the general observation that FAs are low in postmortem brain tissue in LOAD, there is no current agreement on which group or species of the numerous FAs found in brain are most altered in LOAD [4,23].

### 1.10. Overall Design and Hypotheses

We used iPSC lines from participants with LOAD and healthy control (HC) participants and analyzed iPSC-derived astrocytes as a first choice, because they have all relevant cell membranes and organelles, use lipids as signaling molecules, and have a role in inflammation, all of which may be altered in LOAD. They are involved in synapse integrity, protection against excitatory neurotoxicity, and maintenance of the blood–brain barrier [61]. And astrocytes provide precursors for energy metabolism and cholesterol for membrane maintenance to neurons [21]. These properties make them highly relevant to risk for LOAD.

For a general estimate of cell lipids, whole cell lipid content was analyzed. Different organelles have somewhat different lipid constituents [20], and mitochondria-enriched fractions were isolated from the astrocytes, and their membrane contents analyzed. Mitochondria were chosen because their membrane composition can profoundly affect the bioenergetic flux [62], and bioenergetic abnormalities have been observed as prominent features in brains and cells from individuals with LOAD [22,63].

Previous results, in the brain, postmortem, and peripheral sites, in vivo, observed alterations in cholesterol, glycerophospholipids, and their components, as well as in sphingolipids and FAs in association with LOAD [3,4,21,23,25,28,42,45,46,50,52,54,55]. Notably, levels of CEs are low, in vivo, and low levels are predictive of illness. Exploring whether CE levels are inherently abnormal may fairly represent the primary hypothesis.

## 2. Materials and Methods

### 2.1. Subjects

All experiments and procedures were performed in accordance with relevant institutional and federal guidelines and regulations and were approved by the Mass General Brigham Institutional Review Board, with written informed consent provided by all subjects. The subjects (Table 1) were recruited from the McLean Hospital Memory Diagnostic Clinic and diagnosed by a geriatric psychiatrist using the Diagnostic and Statistical Manual of Mental Disorders (DSM-5) criteria [64], using various clinical assessments, including completion of the Montreal Cognitive Assessment (MOCA) test [65]. When clinically indicated, additional neuropsychological testing was conducted to clarify diagnosis. All subjects were assessed for their capacity to provide consent. In the case of incapacity, an authorized guardian/healthcare proxy provided consent, and the subject provided assent.

### 2.2. Tissue Culture

The establishment of the iPSC lines and culture protocols were as described in previous publications [63,66,67]. Fibroblasts or peripheral blood mononuclear cells (PBMCs) donated by subjects were reprogrammed to iPSCs by standard protocols at different facilities, including the Tsai Laboratory at the Picower Institute for Learning and Memory (director, Dr. Li-Huei Tsai); the Human Pluripotent Cell Core Facility, University of North Carolina, School of Medicine, Chapel Hill, NC, USA; New York Stem Cell Foundation (NYSCF), New York, NY, USA; and Cellular Reprogramming Inc., Pasadena, CA, USA. The iPSC lines were produced by Sendai virus or RNA reprogramming methodologies. Generated iPSC were characterized by assessing the expression of pluripotency markers using the Nanostring nCounter^®^ Stem Cell Characterization Panel or TaqMan hPS Score Card Assays, cell morphology, and immunocytochemistry of pluripotent stem cell markers, including octamer-binding transcription factor 4 (OCT4), SRY-box transcription factor 2 (SOX2), tumor-related antigens (TRA)-1-60 and -81, stage-specific embryonic antigen-4 (SSEA-4) or homeobox protein NANOG, in vitro or in vivo differentiation to embryoid bodies, and karyotyping by G-banded metaphase spread or the Nanostring nCounter Plex2 Assay Kit [63,68].

At passage numbers of 18 or higher, the iPSCs were differentiated to NPC and astrocytes, as previously published [63,66,67]. The iPSCs were dissociated to single cells and plated in AggreWell™ 800 microwell culture dishes (StemCell Technologies, Vancouver, BC, Canada, Cat No. 34815) to form embryoid bodies (EBs) in Neural Induction Media (StemCell Technologies, Cat No. 05839) supplemented with 2 μM dorsomorphin (Stemcell Technologies, Cat No. 72102), 10 μM SB431542 (Stemgent, Yokohama, Japan, Cat No. 04-0010), and 0.2 µM LDN193189 (Biogems, Westlake Village, CA, USA, Cat No. 1066208). At day in vitro (DIV) 5, EBs were transferred to vitronectin-coated (VTN, Gibco, Billings, MT, USA, Cat No. A14700) plates and cultured in the same media. Clusters of NPC-containing neural rosettes were observed after DIV 6 and transferred to VTN-coated culture dishes at DIV 12. After growth to confluency, the NPCs were plated at a density of 1–2 × 10^5^ cells/cm^2^ using Accutase™ (Millipore, Burlington, MA, USA, Cat No. A6964) and cultured in Neuro Basal Media (1:1 composition of Neurobasal™ -A media (Gibco, Cat No. 10888-022) and DMEM:F12 (Gibco, Cat No. 12320033), supplemented with 1 × B27 supplement without Vitamin A (Gibco, Cat No. 12587-010), 1 × N2 supplement (Gibco, Cat No. 17502-048), 1 × MEM Non-Essential Amino Acids (MEM-NEAA, Gibco, Cat No. 11140-050), 2 mM GlutaMax, 100 U/mL Penicillin/Streptomycin, 100 µM β-mercaptoethanol (ß-ME, Sigma-Aldrich, St. Louis, MO, USA, Cat No. M3148), 10 ng/mL EGF (R&D Systems, Minneapolis, MN, USA, Cat No. 236-EG, and 10 ng/mL bFGF (Gibco, Cat No. 13256029). The NPCs were passaged every 5–7 days using Accutase and characterized for expression of SRY-box transcription factor 1 (SOX1) (Millipore, Cat No. AB15766) or SOX2 (Abcam, Cambridge, UK, Cat No. ab97959), paired box gene 6 (PAX6) (BioLegend, San Diego, CA, USA, Cat No. 901301), and type VI intermediate filament protein, NESTIN (R&D systems, Cat No. MAB1259), by immunocytochemistry (ICC) at passage between 3 and 5.

To differentiate astrocytes, the NPCs were plated at a density of 1.5 × 10^4^ cells/cm^2^ on VTN-coated plates with NPC media, and the next day, the media was changed to astrocyte media (ScienCell Research Laboratories, Carlsbad, CA, USA, Cat No. 1801), supplemented with 10 ng/mL BMP-4, 10 ng/mL CNTF, and 10 ng/mL Heregulin-β1 (all Peprotech, Thermo Fisher Scientific, Waltham, MA, USA, Cat No. 120-05, 450-13, and 100-03 in order). Astrocytes were grown to confluency and propagated every 5–7 days at a seeding density of 1.5 × 10^4^ cells/cm^2^ using Accutase. The cells were characterized for expression of astrocyte-specific markers, including glial fibrillary acidic protein (GFAP) (Agilent Dako, Santa Clara, CA, USA, Cat No. Z0334), excitatory amino acid transporter 1 (EAAT1) (Alomone Labs, Jerusalem, Israel, Cat No. AGC-021), and calcium-binding protein S100β (Sigma-Aldrich, Cat No. S2532) by ICC at DIV 30.

### 2.3. Mitochondria Extraction

Nine million astrocytes were used for mitochondrial extraction utilizing a Mitochondria Isolation Kit for Cultured Cells (Thermo Fisher Scientific, Cat No. 89874) following the manufacturer’s instruction with slight modifications according to Kappler [69], Preble [70], Williamson [71], and Kurokin [72]. To minimize tthermal effects on mitochondrial integrity, all reagents, instruments, and intermediates of mitochondrial extracts were kept on ice during the entire process, and the temperature of the centrifuge was set at 4 °C. The cells were lysed with Reagent A from the kit, with a brief vortex followed by incubation on ice for 2 min and subsequent homogenization using a KIMBLE Dounce Tissue Grinder (DWK life sciences, Wertheim, Germany, Cat No. 885300-0002). Reagent C from the kit was added to the homogenized cell lysates, and the mixture was centrifuged to remove cellular debris. Astrocyte lysates were centrifuged in two steps, at 1000× *g* for 5 min and 1300× *g* for 10 min. Supernatants containing the cytosolic and mitochondrial-enriched fractions were then partitioned for further processing: about 1/3 for Western blots and 2/3 for lipidomic analysis. The separation into cytosolic fractions, as supernatants, and mitochondria, as pellets, was achieved with 15 min centrifugation at 3000× *g*. After a washing step with Reagent C, mitochondrial pellets for Western blots were solubilized in 2% 3-[(3-Cholamidopropyl)dimethyl-ammonio]-1-propane sulfonate (CHAPS, Roche, Basel, Switzerland, Cat No. 10810118001) dissolved in 1 X Tris-Buffered Saline (TBS, Boston BioProducts Inc., Milford, MA, USA, Cat No. BM-301X) in distilled water, vortexed for 1 min, and centrifuged at 15,000 rpm for 2 min to obtain supernatants as a final mitochondrial fraction. Mitochondria-enriched pellets used for lipidomic analysis were washed with Reagent C and resuspended in 1 X DPBS without magnesium and calcium and stored at −80 °C until further use.

### 2.4. Western Blots

Proteins were quantified by the Pierce bicinchoninic acid (BCA) assay (Thermo Fisher Scientific, Cat No. 23225), with approximately one million astrocytes lysed in RIPA Lysis and Extraction buffer (Thermo Fisher Scientific, Cat No. 89900) used as positive controls. Twenty-five micrograms of whole-cell lysates or mitochondria-enriched extracts were mixed with Western-Ready Protein Sample Loading Buffer (BioLegend, San Diego, CA, USA, Cat No. 426311) containing 5% beta mercaptoethanol (ß-ME, Sigma-Aldrich, Cat No. M3148) and heated at 60 °C for 10 min. The denatured samples were run on 12% sodium-dodecyl-sulfate polyacrylamide gel electrophoresis (SDS-PAGE) gels at 100 V for about 1.5 h. The gels were then blotted on polyvinylidene difluoride (PVDF) membranes (Bio-Rad, Hercules, CA, USA, Cat No. 1620177) at 100 V for 2 h, followed by membrane blocking with 5% skim milk (Bio-Rad, Cat No. 1706404) in 1 X TBS in distilled water with 0.1% TWEEN-20 (Sigma-Aldrich, Cat No. P1379) at RT for 1 h. Several subcellular organelle markers [69] were used to determine the purity of the mitochondria-enriched extracts (Supplementary Table S1, Supplementary Figure S1). Hybridized primary antibodies were captured through horse-radish peroxidase (HRP)-conjugated secondary antibodies (Vector Laboratories, Newark, CA, USA; Cat No. PI-2000-1, RRID: AB_2336177; and Cat No. PI-1000-1, RRID: AB_2336198) at RT for 1 h, followed by treatment with the West-Q Femto Clean electrogenerated chemiluminescence (ECL) solution (GenDEPOT, Katy, TX, USA, Cat No. W3680-020), and signals were measured using a ChemiDoc MP imaging system equipped with Gen5_2.0 software version 2.03 (Bio-Rad, RRID: SCR_019037). The method for isolating mitochondria used here revealed mitochondria-enriched fractions that are deprived of ER and Golgi apparatus but still contain some residuals of smaller subcellular organelles, including (auto)phagosomes or lysosomes (Supplementary Figure S1).

### 2.5. Lipidomics Analysis

A mass spectrometry-based lipid analysis was performed by Lipotype GmbH (Dresden, Germany) as described [73]. The analysis was performed on 10^6^ whole-cell astrocytes and 40–60 µg mitochondrial protein provided to the company. Lipids were extracted using a two-step chloroform/methanol procedure mode [73,74]. Gangliosides were extracted from the water phase of the preceding chloroform/methanol extraction with a solid phase extraction protocol. Samples were spiked with internal lipid standard mixtures as described [73,75,76]. The mixtures contain cardiolipin 14:0/14:0/14:0/14:0 (CL), ceramide 18:1;2/17:0 (Cer), diacylglycerol 17:0/17:0 (DAG), hexosylceramide 18:1;2/12:0 (HexCer), dihexosylceramide 18:1;2/12:0 (DiHexCer), globoside 3 18:1;2/17:0 (Gb3), gangliosides GM3-D3 18:1;2/18:0 (GM3), GM1-D3 18:1;2/18:0 (GM1), lysophosphatidate 17:0 (LPA), LPC 12:0, LPE 17:1, lysophosphatidylglycerol 17:1 (LPG), lysophosphatidylinositol 17:1 (LPI), lysophosphatidylserine 17:1 (LPS), PA 17:0/17:0, PC 15:0/18:1 D7, PE 17:0/17:0, phosphatidylglycerol 17:0/17:0 (PG), PI 16:0/16:0, PS 17:0/17:0, CE 16:0 D7, sphingomyelin 18:1;2/12:0;0 (SM), sulfatide d18:1;2/12:0;0 (Sulf), triacylglycerol 17:0/17:0/17:0 (TAG) and cholesterol D6. After extraction, the organic phase was transferred to an infusion plate and dried in a speed vacuum concentrator. In the first step, the dry extract was re-suspended in 7.5 mM ammonium acetate in chloroform/methanol/propanol (1:2:4; V:V:V), and in the second step, the dry extract was re-suspended in 33% ethanol solution of methylamine in chloroform/methanol (0.003:5:1; V:V:V). All liquid-handling steps were performed using the Hamilton Robotics STARlet robotic platform with the Anti Droplet Control feature for organic solvent pipetting.

The samples were analyzed through direct infusion on a QExactive mass spectrometer (Thermo Scientific) equipped with a TriVersa NanoMate ion source (Advion Biosciences, Ithaca, NY, USA), and analyzed in both positive and negative ion mode, with a resolution of R_m/z=200_ = 280,000 for MS and R_m/z=200_ = 17,500 for MSMS experiments in a single acquisition. MSMS was triggered by an inclusion list encompassing corresponding MS mass ranges scanned in 1 Da increments [77]. Both MS and MSMS data were combined to monitor CE, Chol, DAG, and TAG ions as ammonium adducts; PC and PC O-, as acetate adducts; and CL, PA, PE, PE O-, PG, PI, and PS as deprotonated anions. MS was only used to monitor different forms of glycosphingolipids (Gb3, Gb4, GM4, GM3, GM2, GM1, GD3, GD2, GD1, GT3, GT2, GT1, and GQ1), Sulf, LPA, LPE, LPE O-, LPI, and LPS as deprotonated anions, and Cer, HexCer, DiHexCer, SM, LPC, and LPC O- as acetate adducts and cholesterol as ammonium adduct of an acetylated derivative [78]. Since the MSMS mode was used to characterize acyl chains in the esterified lipids only, the content of free FAs was not directly evaluated. Altogether, individual species of 33 lipid classes were detected (Supplementary Table S2). A detailed description of lipidomic profiling by Lipotype is presented in Liebisch [78] and Surma [77].

### 2.6. Data Processing

Lipid species were identified based on molecular mass using LipotypeXplorer (Lipotype). The identified lipid species were primarily quantified by peak areas, which were filtered by signal-to-noise and signal-to-background ratios to sort out false signals [79]; the lipid species whose ratios were lower than 5 were filtered out. The filtered lipid species in peak areas were then converted to lipid amounts in pmol units using calibration curves established by internal lipid standards. Prior to downstream analysis, data filtering was applied using an occupational threshold of 0.7 [75,76]; that is, lipid species that were measured in more than 70% of either control subjects or LOAD patients were kept in the analysis. Also consistent with standard analyses, missing values, that is, lipid species undetected in individual samples at the thresholds obtainable, were imputed by half the minimum value of detection for each lipid species. Lipid amounts were calculated as mol% of total lipids, and lipid species data were summed within lipid classes. The results from whole cells and mitochondria-enriched fractions were processed independently. Data processing was performed in R (version 4.4.2; RRID: SCR_001905).

### 2.7. Statistics

Unless other stated, two-tailed Students’ *t*-tests, uncorrected for multiple measures, were used to compare measurements of lipids between the samples from individuals with LOAD and healthy control subjects. The analyses were performed in Microsoft Excel for Mac, version 16.96.1, and PRISM 9 for macOS, version 14.1. (GraphPad). The differences were considered statistically significant when *p* values were less than 0.05, while *p* values between 0.05 and 0.1 were considered as a trend towards significance. Lower CE levels, consistently associated with LOAD in CSF and blood studies, were treated as a prior hypothesis. The control subjects included some samples from people who were younger than those in the LOAD group. As noted above, age-related markers are eliminated by reprograming. However, we confirmed this absence of an age effect for CEs, the lipid we hypothesized to be abnormal in association with LOAD. No correlation of the CE level with age was found in either the LOAD-associated (r = 0.17, *p* = 0.61) or control (r = −0.46, *p* = 0.15) samples.

## 3. Results

Filtration of the raw data revealed that 41.7% of whole-cell and 55.3% of mitochondria-enriched lipid species and 11.1% of whole-cell and 19% of mitochondria FAs were filtered out using an occupational threshold of 0.7 and an imputation of half the minimum. In a second analysis, an occupational threshold of 0.8 was used, and the results did not substantially differ from the data with an occupational threshold of 0.7. In general, while data filtering removed individual species, it did not impact the composition of classes or the distribution of FAs. The results are presented for lipid contents in whole-cell preparations or mitochondria-enriched fractions. Differences are noted as percent changes in lipid levels in cell lines derived from individuals with LOAD compared to healthy control subjects. In either cohort, there were no significant effects of subjects’ ages on data outcome. For comparison with results from postmortem, CSF, and plasma studies, the data were analyzed by lipid classes.

### 3.1. Lipid Levels in Whole Cells

After data filtration and imputation (see Section 2.6., data processing), a total of 1047 lipid species were detected in astrocytes, among which 684 (65.3%) were lower and 363 (34.7%) were higher in cells from individuals with LOAD. No prior hypothesis was proposed regarding results at the species level. Rather, as detailed above, the hypotheses were based on prior studies that examined lipids by class. A summary of the results by lipid classes is given in Table 2, and the details of the results are described below.

*Levels of Cholesterol and Cholesterol Esters:* Neutral cholesterol levels were not different in cells derived from individuals with LOAD (103%, SEM = 5%). The levels of CEs, however, were quite different between groups. Five CEs passed the filtration criteria and as a class were 53% lower in the LOAD group than in the cells from the HC group. Reduced CE levels in cells from individuals with LOAD represents a prior hypothesis. Therefore, a one-tailed *t*-test, uncorrected for multiple measures, is appropriate, giving a *p* = 0.021 (with SEM = 15.6%), for the deficit in CE levels observed in these cells. While significantly lower CE levels were observed in CSF and blood of people with LOAD, those studies did not report which species within the lipid class CE were low. Different species of CE have different FA side chains, and these were measurable by the methods applied in this study. Notably, all five detectable CE species were substantially decreased in cells from individuals with LOAD. Decreases for individual species of CE ranged from 49 to 62%, with *p* values ranging from 0.015 to 0.028 (Table 3).

APOE variants may differentially transport CEs. Therefore, evidence of a possible association of APOE4 genotype and CE level was explored in the sample populations. Of the 11 healthy comparison individuals, 2 were heterozygote carriers of APOE4, and one of the patients with LOAD carried an E3/3 haplotype (Table 1). When the groups were clustered by APOE3 or E4 genotype, CE, as a lipid class, was 27% (SEM = 20.2%, *p* = 0.194) lower in the APOE4 carrier group, suggesting that APOE4 may contribute to low CE levels in the cells from people with LOAD. However, as most of the individuals with APOE4 genotypes were individuals with LOAD, this difference may reflect other factors leading to risk for LOAD. Therefore, the results were examined separately within the LOAD and healthy comparison groups. The two samples from the people in the healthy comparison group who carried an E4 haplotype had CE values of 2.19 and 1.34 mol%, averaging 1.76 mol%, which was like the whole healthy control group average of 1.4 mol%. The cells from the individuals with LOAD genotyped as APOE3/3 had CE levels of 0.21 mol%, even lower than the average for the whole group with LOAD, at 0.66 mol%, or the entire group with an E4 haplotype, at 0.88 mol%. Taken together, the data did not suggest an association of APOE4 genotype with lower CE levels. Rather, it suggested a direct association of low CE levels with LOAD.

*Glycerophospholipids and their Derivatives:* Glycerophospholipids in astrocytes derived from iPSC did not follow the patterns observed in postmortem studies. Contrary to the decreases observed in postmortem brain analyses of LOAD, PA appeared to be substantially elevated, by 253% (SEM = 106.2%), in the LOAD group versus the HC group, with *p* = 0.029 for the difference. LPA was also elevated, by 83% (SEM = 37.2%), trending towards significance at *p* = 0.098. PC was detected with and without etherized side chains. Both classes were modestly, but non-significantly, decreased. LPC was increased 42%, and LPC with an etherized side chain was decreased by 13%, with neither difference reaching statistical significance. The ratio of un-etherized LPC/PC was 43.5% and the ratio of total LPC/PC was 29% higher in the LOAD samples compared to controls, but these data did not reach statistical significance (SEM = 46.9%, *p* = 0.4 and SEM = 38.8%, *p* = 0.51, respectively). The ratios of un-etherized or total LPE/PE were also not significantly different between HC- and LOAD-associated astrocytes (101.7%, SEM = 21%, *p* = 0.95 and 89.8%, SEM = 19.1%, *p* = 0.72, respectively). DAG appeared to be decreased in the LOAD group (down 14%, SEM = 7.5%) versus the healthy comparison group, and the difference was not significant (*p* = 0.174). This decrease in DAG is opposite to increases observed in studies of postmortem brains and plasma in people with LOAD [23,33,49]. TAG was also slightly reduced, by 8%, but that deficit was not statistically significant (*p* = 0.833).

*Sphingolipids:* Neither ceramides, which show changes over the course of illness in postmortem studies of LOAD, nor sphingomyelin appeared to be much different between cells derived from individuals with LOAD and cells from healthy controls. There were no differences approaching nominal significance nor even notable trends toward group differences in sphingolipids.

**Figure 1:**
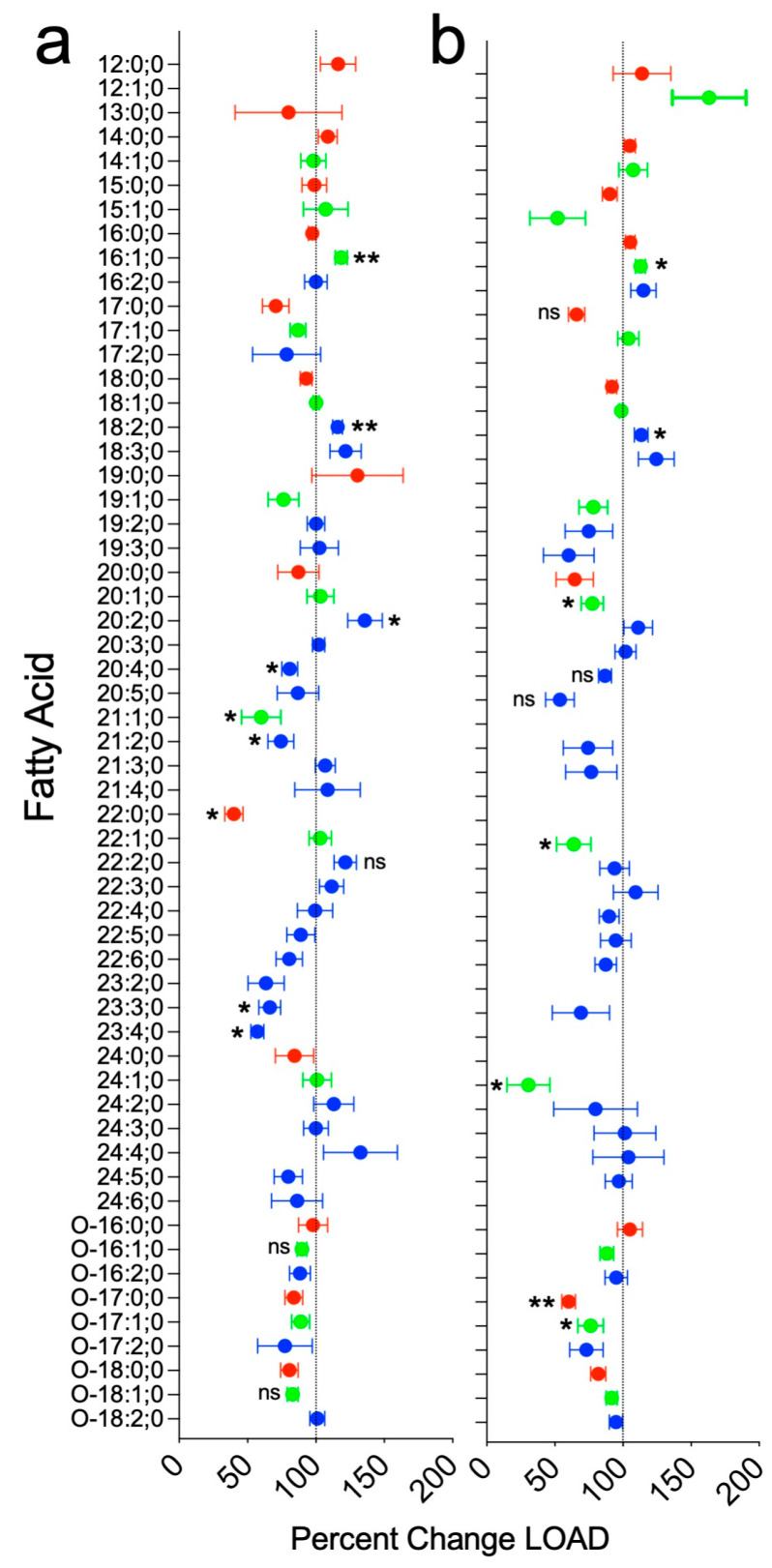
Fatty Acids. FA in (**a**) whole cell astrocyte preparations or (**b**) isolated mitochondria-enriched preparations plotted as percent lipid levels in LOAD over healthy control associated samples using mol% values. Red: Saturated FAs; green: Monounsaturated FAs; blue: Polyunsaturated FAs. Data were analyzed by two-tailed *t*-tests. FA levels are labeled where the difference between values in LOAD- and HC-associated samples reached a trend towards significance *p* < 0.1 (ns), or significance, uncorrected for multiple measures, *p* < 0.05 (*), or *p* < 0.02 (**). Unlabeled differences did not reach a *p* value less than 0.1.

*Fatty Acids*: Individual FAs were more likely to be decreased in cells derived from the individuals with LOAD (see Figure 1a). For the entire group, the reduction averaged 6% (SEM = 7.3%, *p* = 0.44). Of 56 species detected, 14 (25%) were saturated FA, and these were decreased by an average of 9% (SEM = 9.4%, *p* = 0.273) in the cells in the LOAD group. The largest decrease was seen for docosanoic acid (22:0), which was reduced by 60% (SEM = 6.7%, *p* = 0.046). There were 13 monounsaturated FAs (23% of all FA species detected), and as a group, they were decreased by 6%, (SEM = 3.9%, *p* = 0.39). The greatest decrease observed was in heneicosenoic acid (21:1), which was reduced by 40% (SEM = 14.4%, *p* = 0.049), while palmitoleic acid (16:1) was significantly increased by 18.5% (SEM = 4.4%, *p* = 0.01). There were 29 polyunsaturated FAs (52% of all species detected), and, as a group they were decreased by 4% (SEM = 3.8%, *p* = 0.352). Among the polyunsaturated FA species, several were substantially reduced, including two members of the docosatetraenoic acid group: 23:4, decreased by 43% (SEM = 4.7%, *p* = 0.020), and 23:3, decreased by 34% (SEM = 8%, *p* = 0.035). Arachidonic acid (20:4), which plays a role in inflammation [80], was decreased by 19% (SEM = 5.8%, *p* = 0.027) in the LOAD group. One polyunsaturated FA, linoleic acid (18:2), was significantly increased, by 15.8% (SEM = 3.6%, *p* = 0.008). More details on the trends in individual species, many of which are decreased in the LOAD group, are shown in Figure 1a.

### 3.2. Lipid Levels in Mitochondria-Enriched Fractions

In mitochondrial fractions isolated from astrocytes, a total of 448 lipid species were detected after data filtration and imputation, of which 341 were lower (76.1%) and 107 (23.9%) were higher in samples from individuals with LOAD. As with the whole-cell results, lipid species were grouped by class (Table 4).

Mitochondrial membranes have a more restricted composition than other cell membranes, and not all the lipids and differences observed in mitochondria are the same as those observed in whole-cell preparations.

*Levels of Cholesterol:* As observed in the results from analyzing whole-cell astrocytes, cholesterol was not different in mitochondria-enriched fractions isolated from cells derived from people with LOAD and HC individuals (13% increase, SEM = 4.1%, *p* = 0.642). Most CEs are stored in separate organelles, and the low amounts of CE in the mitochondria isolations were below the level of detection in the Lipotype assays.

*Glycerophospholipids and their Derivatives:* The only glycerophospholipid that had substantially different levels in mitochondria-enriched fractions from the cells derived from the people with LOAD was PG, which was decreased by 23% (SEM = 4.7%, *p* = 0.006).

Among other glycerophospholipids, most showed no substantial difference between groups. However, lysoglycerophospholipids were generally lower in mitochondrial fractions from individuals with LOAD. All lysoglycerophospholipid types were lower, by 50–60%, with LPC reduced by 53% (SEM = 5.8%, *p* = 0.096). The ratios of un-etherized LPC/PC or total LPC/PC were down by 54% (SEM = 6.13%, *p* = 0.1) and 46% (SEM = 12.9%, *p* = 0.19), respectively. The un-etherized or total LPE/PE ratios were also decreased in LOAD-associated mitochondria-enriched fractions, by 45% (SEM = 4.86%, *p* = 0.08) and 46.5% (SEM = 5.1%, *p* = 0.13), respectively. Unlike the results from the whole-cell preparations, DAG was not decreased in mitochondria isolations from the LOAD group.

*Sphingolipids:* Ceramides in mitochondria-enriched preparations from the LOAD group trended low, being decreased by 23% for the entire class, but these differences did not reach statistical significance (*p* = 0.204). However, adding to the suggestion of a deficit, there were six species of ceramide detected, each with a different fatty acid side chain, and every species was lower in the LOAD group. Although none of these deficits reached statistical significance, the finding that they were all reduced suggests the possibility of a deficit in ceramides in mitochondria in LOAD.

*Fatty Acids*: Fewer species of FAs were detected in mitochondria-enriched fractions than in whole cells, but the overall patterns of differences in FA species between groups in the mitochondria preparation were quite like the differences seen in the whole-cell preparations, see Figure 1b. Thus, as in the whole cells, FAs, on average, were decreased by 10% (SEM = 3.4%, *p* = 0.278), in the LOAD group. Among 47 FA species detected, there were 10 (21%) species of saturated FAs. Most were reduced in the LOAD group, and the whole group was down by 12% (SEM = 8.1%, *p* = 0.30). The highest significant difference was reached for heptadecanoic acid (17:0), down by 34% (SEM = 6.0%, *p* = 0.032), and its etherized form decreased by 40% (SEM = 5.0%, *p* = 0.088). There were 13 (28%) monounsaturated species detected, which were decreased by 12% on average for the whole group (SEM = 9.6%, *p* = 0.265). Individual monounsaturated species with decreases that were statistically significant or nearly significant included eicosenoic acid (20:1), which was decreased by 23% (SEM = 8.2%, *p* = 0.047), heptadecenoic acid ether (0–17:1), which was decreased by 24% (SEM = 9.4%, *p* = 0.055), docosenoic acid (22:1), which was decreased by 36% (SEM = 13%, *p* = 0.063), and nervonic acid, named that way because it was first discovered in mammalian nervous tissue, which is essential for brain health [81] (24:1), which was decreased by 69% (SEM = 16%, *p* = 0.079). One monounsaturated species, palmitoic acid (16:1) was increased by 13% (SEM = 3.6%, *p* = 0.017), and dodecenoic acid (12:1) was increased by 63% (SEM = 27%, *p* = 0.159) in the LOAD group. There were 24 polyunsaturated FAs (51% of all species). On average, they were decreased by 9% (SEM = 9.4%, *p* = 0.46). Most individual species were decreased, but none of these decreases reached statistical significance. The greatest deficit was observed in eicosapentaenoic acid (20:5), an omega-3 fatty acid involved in inflammation [82]. It was reduced by 46% (SEM = 10.1%, *p* = 0.091). There was a trend towards an increase in linoleic acid (18:2) by 13.3% (SEM = 5%, *p* = 0.032).

## 4. Discussion

### 4.1. Context of the Studies

Findings regarding postmortem tissue, along with results from CSF and plasma studies, reflect changes associated with progression and treatment of LOAD, as well as aging and any associated illness. Postmortem studies often represent disease endpoints. Results from studies of reprogrammed cells, lacking markers of aging or disease, as reported here, are complementary to postmortem, CSF, and plasma studies in that they can detect inherent factors. Taken together, clinical studies and reprogrammed cell culture studies may both identify and separate mechanisms underlying neuropathological risk factors and progression of illness.

To our knowledge, no previous studies have determined whole-lipid contents in cells obtained from people with LOAD reprogrammed to iPSCs and then differentiated to become brain cells. We believe that these are the first results that provide such data for comparison with other approaches determining lipid levels associated with LOAD.

There are a few studies using isogenic APOE4 iPSC lines that study the relationship of APOE variants with some aspects of lipid metabolism [83,84,85,86]. Generally, these are not studies of lines from people with LOAD. Nevertheless, they are worth noting. One past study observed that iPSC-differentiated APOE4 isogenic astrocytes had a 20% increase in de novo synthesis of total cholesterol but not CE, as well as increased cholesterol accumulated in lysosomes when compared to the same APOE3 haplotype cell line [83]. Other studies on isogenic APOE4 iPSC lines confirm that APOE4 is associated with altered metabolism of cholesterol and FAs, as well as with increased formation of lipid droplets [84,85]. In a study of iPSC from individuals with LOAD differentiated to cortical neurons, dysregulation of genes involved in lipid metabolism was observed, but lipid levels were not measured [86]. Further work combining characterization of lipid levels, as performed here, and of underlying mechanisms, including the role of APOE4, should lead to the identification of specific aspects of lipid composition and lipid metabolism that increase the risk for LOAD.

Past in vivo or postmortem results are detailed in the Introduction. Briefly, past studies observed no change in cholesterol but did observe lower cholesterol ester levels in the brain, postmortem, CSF, and plasma of individuals with LOAD [24,25]. Most glycerophospholipids have been observed to be decreased in brain in association with LOAD, but with LPCs or LPEs elevated in the brain and low in plasma [3,4,21,42,44,45,46,47,48]. PGs and DAGs were also observed to be elevated in the brain [4,23,33,49]. Abnormalities in sphingolipids, postmortem, appeared to vary between studies and with length of illness [3,4,23,28,42,50,52]. FAs were generally observed to be low, postmortem, but there was no consensus on which specific species were most abnormal [4,23,53,54,55]. Here, we observe which of these abnormalities could be inherent to astrocytes from people with LOAD, as observed in the reprogrammed cell lines. These abnormalities may underlie the risk for LOAD rather than being consequences of disease progression and treatment.

### 4.2. Review of the Findings

The findings regarding substantial deficits of CEs in astrocytes from people with LOAD are consistent with similar abnormalities reported in vivo in blood and CSF studies (see [21,24,31,32,34]). By comparison, the levels of cholesterol, per se, were not substantially or statistically significantly different in astrocytes from people with LOAD and HC. Similarly, in clinical studies of LOAD, no substantial abnormalities of cholesterol levels were observed [24]. Also, a recent meta-analysis of the effects of cholesterol on AD in combination with a meta-analysis of cholesterol serum levels revealed no association of high-density lipoprotein cholesterol (HDL-C,) total cholesterol, and TG with AD but a significant impact of low-density lipoprotein cholesterol (LDL-C) on the development of AD [87]. It is possible that the modest decreases in cholesterol observed here, in cells from people with LOAD, are related to some deficiency in cholesterol synthesis noted in clinical studies of LOAD [15,16] or in studies on postmortem brain tissue using targeted metabolomics and gene expression profiling that suggested decreased de novo cholesterol synthesis in response to impaired cholesterol catabolism and efflux [25]. Of note, astrocytic synthesis and release of cholesterol is important for maintaining brain cholesterol homeostasis and particularly its transport to neurons is essential for neuronal cholesterol homeostasis [88]. Thus, a reduction in de novo cholesterol biosynthesis in astrocytes, as suggested from our study, indicates that disturbed brain cholesterol homeostasis could be an inherent risk factor for LOAD.

Contrary to findings from postmortem studies, as reviewed above, substantial decreases in PC or most other glycerophospholipids were not seen, with the exception that PG was low both in whole-cell astrocyte preparations and mitochondria-enriched fractions. PA, reported along with other glycerophospholipids to be decreased in postmortem studies of LOAD [3,21], was increased in whole-cell preparations, though not in the mitochondria preparations, in the samples from people with LOAD studied here. Evidence that the ratio of LPC/PC was abnormal, and even opposite in whole cells versus mitochondria, was notable but inconclusive.

It may not be surprising that major lipids, specifically cholesterol and glycerophospholipids that are present at high concentrations and are used structurally in cell membranes, were similar in the two groups. Large changes in cholesterol and some glycerophospholipids, such as PC, would greatly compromise many, if not most, cell functions, and might be expected to be associated with substantial developmental disorders or the early onset of neurodegeneration. Therefore, even if there are genetically determined abnormalities in the metabolism of these classes of lipids, there may also be compensatory mechanisms that maintain the lipids at roughly normal levels, at least in early life. The findings of large changes in glycerophospholipids in postmortem studies could be associated with cell disintegration, as occurs in established disease.

While major lipids are largely structural, CE is a signaling and storage molecule, present at levels an order of magnitude lower than those of neutral cholesterol, and the effects of abnormalities in CE levels may be better tolerated, causing less dysfunction in cell processes. Similarly, abnormal levels and metabolism of a few glycerophospholipids, such as PG or PA, as seen here, or in LPC/PC, might be better tolerated than deficits in all glycerophospholipids as a class, as observed postmortem in LOAD [24]. Of note, the finding that LPCs and LPEs but not PCs and PEs were increased in whole-cell astrocytes and decreased in the mitochondria-enriched fraction could indicate a cellular disbalance of lysoglycerophospholipid synthesis with an overproduction or accumulation in the cytoplasm and a deficit in mitochondria.

Nonetheless, CEs are necessary, as a precursor, for maintaining cholesterol in cell membranes, and altered cholesterol precursor levels found in CSF and plasma of patients has suggested that cholesterol de novo synthesis is reduced in LOAD [16]. CE levels decrease naturally with age [89], and inherently low CE as seen in cells from patients with LOAD might be a risk factor for inadequate membrane maintenance over the course of a long life. In fact, CEs may play a key role in a cascade of effects underlying illness progression [23]. Specifically, in vitro studies observed that higher CE levels are associated with a decreased production of toxic Aβ oligomers and lower CE with increased synaptic damage from Aβ fragments [90,91,92]. And decreased production of cholesterol, typically dependent on the levels of CEs, increases hyperphosphorylation of Tau protein [93]. By these mechanisms, the lower CE levels observed here in the cells from people with LOAD may directly contribute to the production and effects of toxic abnormal proteins seen in many cases of LOAD.

FAs of all subtypes were consistently, though modestly, reduced in the LOAD group studied here, in both whole cells and mitochondria-enriched isolations. This is consistent with results from clinical studies of FAs in association with LOAD [4,53,55]. Some species were reduced more than others. Only a few were elevated. Overall, the findings suggest imbalances in FA content associated with LOAD. As with CEs, these reductions and imbalances could compromise many cell functions but might well be tolerated during development and early life, only leading to an increased risk of illness over time. Abnormal concentrations of FAs in glycerophospholipids would affect cell membrane fluidity [21] and thereby alter the processing of proteins, including amyloid precursor protein (APP). Membrane fluidity abnormalities have been proposed elsewhere as a mechanism underlying the production of toxic Aβ oligomers [94]. In addition, FAs in astroglia cells can activate the production of proinflammatory molecules causing chronic neuroinflammation in the brain [95]. As noted, FAs, including VLCFAs in connection with peroxisomes, can also serve as stores of substrates for energy production through β-oxidation, and alterations of β-oxidation, loss of peroxisomes, or peroxisomal dysfunction can cause oxidative stress, chronic inflammation, and an accumulation of Aβ, which are all pathological mechanisms in those at risk for LOAD [59,60]. Our observation of a tendency of VLCFA levels to be reduced in astrocytes of people with LOAD does not necessarily indicate inherent impairment of peroxisome functions. However, this possibility merits further investigation. It is important to note again that the fatty acids observed in our study are not free fatty acids (FFAs); they are bound to backbones, such as glycerophospholipids or sphingolipids. They can be released to become FFAs and be modified as part of inflammatory or other cell-signaling processes. Thus, abnormal levels of these bound FAs may indicate downstream compromise in these cell processes.

What may explain these inherent differences in cell lipid contents? There may be abnormalities of anabolism and/or catabolism. The ER has been noted as the site where proteins are processed and where APP is misprocessed. And the ER is where lipid droplets, containing CEs and FAs, are produced [7]. Abnormal internal membrane proportions [96], particularly in the ER [97], have been reported in LOAD. Lower CE content might be a consequence of abnormal ER function. In addition, lipids are key components of phagosomes, endosomes, and lysosomes, thus contributing to the processing, digestion, and elimination of proteins and lipids by endocytosis and autophagy [7]. Reciprocal interactions have been observed between lipids and Aβ peptides in LOAD (and EOAD), and these interactions involve the formation and activity of cellular organelles [98,99,100]. This would include mitochondria, as noted. Inherent abnormalities of bioenergetics have been documented in LOAD [22,63]. Altered endocytosis and autophagy have also been observed as inherent properties of cell lines from individuals with LOAD [101,102]. Overall, the evidence suggests that abnormalities of both anabolism and catabolism of lipids may be inherent factors underlying risk for LOAD.

In his original paper on a case of dementia, Alois Alzheimer noted lipid accumulations [103]. This observation has been consistently confirmed in subsequent research on both EOAD and LOAD, with a specific increase in lipid droplets and some of their contents [7,28]. By comparison, in the reprogramed cell lines studied here, decreases in CEs and FAs, both major contents of lipid droplets, were observed. Phosphatidic acid, a precursor in the synthesis of lipid droplet-containing diacyl- and triacylglycerols, was observed to be increased. DAG, and possibly TAG, were observed to be decreased, suggesting a metabolic abnormality at the step from PA to DAG and TAG. The relationship between these inherent lipid deficits and the lipid deficits and accumulations observed post-mortem requires further study.

### 4.3. Limitations of the Current Study and Opportunities for Future Work

Future studies with a greater number and diversity of cell lines are needed to confirm, refute, or expand this work. Other cells, including neurons, may not show the same abnormalities as astrocytes. Lines from more study participants are needed. This includes increasing the number of male and female participants, so that sex differences can be explored. The prevalence of LOAD is higher in women than men, which may be the result of hormonal influences, as well as sex-related differences in inflammatory activity [104]. However, peripheral lipid metabolism differs in men and women, and this may contribute to risk for LOAD, as well [104]. It is unknown if there are inherent differences in lipid metabolism in brain cells from men and women.

Although APOE variants were not clearly associated with the abnormalities observed in the lines from individuals with LOAD studied here, more studies are required to address the importance of APOE haplotypes on lipid content. Future studies will also be needed to determine whether the abnormalities seen here in astrocytes are also seen in other cell types.

Whole-cell extracts contain lipids from a mix of membranes and organelles with different lipid compositions [20]. The proportion of membranes in cells from people with LOAD may differ from those without LOAD. Specifically, the ratio of ER to other membranous elements may be higher in cells from individuals with LOAD [94]. This could affect the results observed in the whole cell preparation.

CEs largely exist in lipid droplets, and it needs to be determined if lipid droplet amounts are increased or decreased in the LOAD cell populations analyzed here. Whether abnormal amounts or functions of the ER, specifically its production of lipid droplets, are related to abnormal levels of lipids observed here will also require further studies.

Mitochondria have an inner and an outer membrane, but the results reported here are related to mitochondria as a whole. Cardiolipin, a lipid that is uniquely found in the inner mitochondrial membrane, has been implicated in mitochondrial dysfunction and brain disorders [105]. In our study, CL was not detected in the mitochondria-enriched fractions and only sporadically detected in a few whole-cell astrocyte samples (Supplementary Figure S2A). While our final analysis criteria filtered CL out, the analysis of the raw data with an occupational threshold of 0.3 revealed three CL species that are slightly increased in LOAD astrocytes (Supplementary Figure S2B,C). Relevant to our study, CL has been detected by shotgun lipidomics in mitochondria isolated from in vivo mouse astrocytoma or in a mouse astrocytoma cell line, in vitro [106]. In a study on human iPSC-derived whole astrocytes, also using shotgun lipidomics, CL was not measured [107]. Of note, the calculated amounts of CL in our iPSC-derived astrocytes were in a range of 0.20 ± 0.14 pmol/µg protein. This is lower than reported for mitochondria extractions in the literature, which range from 10 to 57 pmol/µg protein [69,106,108]. We conclude that, given the low mitochondria input material in our study, CL was below the detection limits of the procedures used by Lipotype.

Overall, mitochondrial lipid composition in the astrocytes from patients with LOAD appears to be abnormal, but future studies will be needed to define abnormalities specific to various mitochondrial lipids and membranes.

Lipids in cells in culture come in part from the growth medium and in part from cellular metabolism of the nutrients the cells receive. Abnormal lipid levels observed in cells from people with LOAD may reflect differences in uptake of nutrients, synthesis of new lipids, or catabolism of lipids. All these mechanisms are ripe for future study.

Lipids are dynamic elements of cells. Profiling lipid levels does not document lipid metabolism and interactions. However, the observation of abnormalities can suggest leads for mechanistic studies. For example, the results reported here would suggest follow-up studies of the formation, interactions, and metabolism of CE in LOAD. Agents that alter CE metabolism and levels in blood are available [109]. Drugs could be developed to modify CE levels and activities in brain. The same might be true for other abnormalities observed here. And lipids, themselves, can serve as restorative or therapeutic agents [110].

Currently, tests to detect risk for AD, or for use in the diagnosis of AD, largely focus on determining Aβ and hpTau or their derivatives in plasma [111]. These tests may be useful, especially for early diagnosis of those cases of LOAD associated with higher levels of plaques and tangles. However, the appearance of these compounds in blood may be secondary to other, more basic, predisposing mechanisms, and elevated levels of Aβ and hpTau in plasma or CSF may only be detectable after damage has already occurred in brain. In addition, many cases of LOAD are not associated with pronounced plaques and tangles, and many people who age without substantial cognitive deficits have high levels of plaques and tangles [112]. Defining inherent cellular processes that contribute to early pathological processes and underlie the appearance of LOAD rather than later stage elements of plaques and tangles may lead to tests that can be applied long before brain damage has occurred. In addition, specific abnormalities observed in those tests, which may differ among individuals at risk, may detect subtypes of LOAD and suggest personalized approaches for interventions, including interventions targeting specific lipids and membranes [22,66,113]. As an example from the results reported here, abnormalities of CE levels and synthesis, or processes underlying abnormalities of lipid droplet formation and contents, may be targets for beneficial modulation that could occur years or decades before the development of LOAD.

## 5. Conclusions

As reviewed, genomic, epidemiologic, clinical, and postmortem studies all observe a link between lipids and LOAD. Studies of astrocytes from people with LOAD, as performed here, can identify which abnormalities, in which specific lipids, are inherently abnormal and may be risk-determining precursors for LOAD. The evidence from these new studies on astrocytes suggests that there are no prominent inherent abnormalities in levels of membrane structural lipids, including glycerophospholipids, sphingolipids, and cholesterol, associated with LOAD. Rather, the inherent abnormalities are in levels of storage and signaling lipids, most prominently CEs, along with imbalances of FAs.

The findings strongly suggest that further study of inherent lipid abnormalities associated with LOAD will be fruitful. They must be replicated and extended to include more cell lines. They should be complemented by studies of mechanistic factors, such as specific metabolic reactions or aspects of lipid transport, that underlie inherent abnormal cell lipid composition associated with LOAD. And they should be complemented by further studies to evaluate how abnormal lipid composition affects cell functions.

Such studies should lead to greater understanding of mechanisms contributing to the risk for LOAD and help explain the development of the specific pathology observed in the brain in LOAD. Identifying key inherent factors associated with the risk for LOAD may lead to screening tests and early interventions that reduce the risk of dementia later in life.

## Figures and Tables

**Table 1:** Subject demographics.

	HC	LOAD
Number of Subjects	11	11
Age (Yrs)	52.1 +/− 21.8	72.9 +/− 9.5
Sex	Male: 8, Female: 3	Male: 6, Female: 5
APOE	E2/2: 1	E3/3: 1
	E2/3: 1	E3/4: 8
	E3/3: 7	E4/4: 2
	E3/4: 2	
MOCA Score	-	13.4 +/− 7.0

HC: Healthy control; LOAD: late-onset Alzheimer’s disease; APOE: apolipoprotein ε; MOCA: Montreal Cognitive Assessment.

**Table 2:** Lipid classes detected in whole-cell astrocyte preparations.

Lipid Class	HC	SEM	LOAD	SEM	% LOAD	% SEM	*p* Value
CE	**1.40**	**0.26**	**0.66**	**0.22**	**47.02**	**15.59**	**0.021**
Cer	0.51	0.04	0.54	0.05	105.94	10.78	0.660
Chol	26.53	1.38	27.32	1.33	102.97	5.02	0.686
DAG	3.49	0.24	2.99	0.26	85.73	7.45	0.174
DiHexCer	0.09	0.01	0.13	0.02	149.31	25.39	0.108
GM3	0.50	0.09	0.55	0.10	109.69	18.88	0.714
HexCer	0.37	0.03	0.38	0.03	104.49	8.32	0.708
LPA	**0.06**	**0.02**	**0.11**	**0.02**	**182.91**	**37.18**	**0.098**
LPC	0.21	0.04	0.30	0.10	141.52	47.15	0.422
LPC O-	0.06	0.01	0.05	0.01	86.82	20.66	0.650
LPE	0.25	0.04	0.26	0.05	103.87	20.23	0.886
LPE O-	0.26	0.07	0.19	0.04	73.34	16.69	0.386
LPG	0.02	0.00	0.01	0.00	87.18	8.16	0.279
LPI	0.05	0.02	0.05	0.01	93.33	23.92	0.882
LPS	0.08	0.02	0.07	0.01	91.18	15.50	0.779
PA	**0.42**	**0.07**	**1.48**	**0.44**	**352.71**	**106.17**	**0.029**
PC	31.63	1.09	30.33	1.21	95.89	3.81	0.434
PC O-	4.64	0.46	4.45	0.36	95.88	7.68	0.745
PE	7.54	0.31	7.87	0.25	104.45	3.35	0.413
PE O-	6.53	0.40	5.74	0.25	87.93	3.81	0.111
PG	1.17	0.10	1.00	0.08	86.05	6.64	0.204
PI	4.29	0.45	4.99	0.16	116.55	3.79	0.157
PS	7.48	0.44	7.83	0.38	104.64	5.12	0.558
SM	2.27	0.09	2.53	0.16	111.76	7.11	0.167
TAG	0.16	0.03	0.15	0.05	91.66	33.65	0.833

Numbers depict average mol% (HC and LOAD) and percent levels in LOAD- over HC-associated samples (%LOAD). *p* values are calculated through two-tailed *t*-tests, except for CEs, for which a one-tailed *t*-test was used. Statistically significant (*p* < 0.05) or trend data (*p* < 0.1) are marked in bold and gray. HC: healthy controls; LOAD: late-onset Alzheimer’s disease; SEM: standard error of the mean.

**Table 3:** CE in whole-cell astrocyte preparations.

CE	HC	SEM	LOAD	SEM	% LOAD	% SEM	*p* Value
16:0;0	**0.28**	**0.05**	**0.14**	**0.05**	**51.33**	**16.61**	**0.028**
16:1;0	**0.15**	**0.03**	**0.07**	**0.03**	**46.62**	**17.04**	**0.024**
18:0;0	**0.14**	**0.03**	**0.05**	**0.02**	**38.12**	**12.13**	**0.015**
18:1;0	**0.72**	**0.14**	**0.34**	**0.12**	**46.81**	**16.12**	**0.026**
18:2;0	**0.12**	**0.02**	**0.06**	**0.02**	**49.17**	**16.87**	**0.026**

Numbers depict average values in mol% (HC and LOAD) and percent lipid levels in LOAD- over HC-associated samples (%LOAD). *p* values are calculated through one-tailed *t*-test. Statistically significant (*p* < 0.05) or trend data (*p* < 0.1) are marked in bold and gray. HC: healthy controls; LOAD: late-onset Alzheimer’s disease; SEM: standard error of the mean.

**Table 4:** Lipid classes detected in astrocyte mitochondria-enriched preparations.

Lipid Class	HC	SEM	LOAD	SEM	% LOAD	% SEM	*p* Value
Cer	0.26	0.04	0.20	0.02	78.62	5.81	0.204
Chol	31.17	1.41	32.07	1.29	102.89	4.13	0.642
DAG	2.74	0.38	2.83	0.18	103.00	6.57	0.848
GM3	0.88	0.15	1.03	0.15	116.99	17.10	0.489
HexCer	0.35	0.04	0.32	0.03	90.68	8.03	0.506
LPC	**0.12**	**0.04**	**0.05**	**0.01**	**46.60**	**5.84**	**0.096**
LPE	0.14	0.04	0.08	0.01	58.41	5.02	0.141
LPE O-	**0.07**	**0.02**	**0.03**	**0.00**	**43.09**	**3.54**	**0.088**
PA	0.09	0.01	0.11	0.03	121.69	33.25	0.535
PC	31.81	1.02	31.85	0.65	100.15	2.05	0.969
PC O-	4.83	0.47	4.87	0.29	100.81	6.05	0.945
PE	8.03	0.50	8.42	0.28	104.90	3.47	0.499
PE O-	6.99	0.42	6.04	0.45	86.43	6.44	0.141
PG	**1.09**	**0.06**	**0.84**	**0.05**	**76.98**	**4.66**	**0.006**
PI	2.37	0.12	2.30	0.11	97.12	4.62	0.675
PS	6.01	0.25	5.94	0.32	98.79	5.29	0.858
SM	3.06	0.17	3.01	0.13	98.55	4.40	0.840

Numbers depict average mol% values (HC and LOAD) and percent levels in LOAD- over HC-associated samples (%LOAD). *p* values are calculated through two-tailed *t*-test. Statistically significant (*p* < 0.05) or trend data (*p* < 0.1) are marked in bold and gray. HC: healthy controls; LOAD: late-onset Alzheimer’s disease; SEM: standard error of the mean.

## Data Availability

The data and materials are made available upon request.

## Supplementary Materials

The following supporting information can be downloaded at: https://www.mdpi.com/article/10.3390/cells15060549/s1. References [114,115] are cited in the supplementary materials.

## Abbreviations

The following abbreviations are used in this manuscript:

Aβ	amyloid beta
APOE	apolipoprotein ε
APP	amyloid precursor protein
CEs	cholesterol esters
CSF	cerebrospinal fluid
DAG	diacylglycerol
DHA	docosahexaenoic acid
DSM-5	Diagnostic and Statistical Manual of Mental Disorders
EOAD	early-onset Alzheimer’s disease
ER	endoplasmic reticulum
FAs	fatty acids
FICC	fluorescence immunocytochemistry
HC	healthy control
iPSC	induced pluripotent stem cells
LDL	low-density lipoprotein
LOAD	late-onset Alzheimer’s disease
LPC	lysophosphatidyl choline
MCI	mild cognitive impairment
MOCA	Montreal Cognitive Assessment
NPC	neural progenitor cell
PA	phosphatidic acid
PC	phosphatidylcholine
PE	phosphatidylethanolamine
PG	phosphatidyl glycerol
PI	phosphatidylinositol
PBMC	peripheral blood mononuclear cells
PS	phosphatidylserine
TAG	triacylglycerol
