# Supplementary material for: Inherent Lipid Composition Abnormalities in Astrocytes Associated with Late-Onset Alzheimer’s Disease (LOAD)
Source: Cells. 2026 Mar 19;15(6):549. doi: 10.3390/cells15060549 (PMC13025802; doi:10.3390/cells15060549)
Supplement: Supplementary file 1 [file cells-15-00549-s001.zip › cells-4063998-supplementary.pdf]

Inherent Lipid Composition Abnormalities in Astrocytes Associated with Late-Onset Alzheimer's Disease (LOAD)

Bruce M. Cohen [1,*], Eunjung Koh [1,2], Kandice R. Levental [3], Ilya Levental [3] and Kai-Christian Sonntag [1,2]

[1] Program for Neuropsychiatric Research, McLean Hospital, 115 Mill St., Belmont, MA 02478, USA; bcohen@mclean.harvard.edu
[2] Laboratory for Translational Research on Neurodegeneration, Program for Neuropsychiatric Research, McLean Hospital, 115 Mill St., Belmont, MA 02478, USA; ekoh@mclean.harvard.edu; kai@mclean.harvard.edu
[3] Department of Molecular Physiology and Biological Physics, University of Virginia School of Medicine 200 Jeanette Lancaster Way, Charlottesville, VA 22903, USA; krl6c@virginia.edu; il2sy@virginia.edu
* Correspondence: bcohen@mclean.harvard.edu; Phone: 1-617-855-3227

Supplementary Material

Supplementary Table S1. Antibodies

Name	Marker	Size (kDA)	Dilution	Hybridization	Catalog number (Provider)	RRID
ATP5A	Mitochondria	55	1:2000	Overnight at 4 ºC	ab14748 abcam	AB_301447
Histone H3	Nucleus	17	1:1000	One hour at RT followed by overnight at 4 ºC	9715S Cell Signaling Technology, MA, USA	AB_331563
ß-actin	Cytosol	43	1:2000	Overnight at 4 ºC	sc-47778 Santa-Cruz Biotechnology, TX, USA	AB_626632
Calnexin	Endoplasmic reticulum	90	1:500	One hour at RT followed by overnight at 4 ºC	sc-23954 Santa-Cruz Biotechnology	AB_626783
GM130	Golgi	140	1:2000	One hour at RT followed by overnight at 4 ºC	12480S Cell Signaling Technology	AB_2797933
LAMP2	Lysosome	105-110	1:8000	Overnight at 4 ºC	354302 BioLegend	AB_11204081
LC3B	Autophagosome	16 & 18	1:2000	Overnight at 4 ºC	ALX-803-080-C100 Enzo	AB_2051773

RRID: Research Resource Identifiers

Supplementary Table S2. Lipid Classes. D: Detected but filtered out; V: Detected and included in the analysis

Abbreviation	Full Name	Whole Cells	Mitochondria
**CE**	Cholesteryl ester	V	△
**Cer**	Ceramide	V	V
**Chol**	Cholesterol	V	V
**DAG**	Diacylglycerol	V	V
**DiHexCer**	Dihexosyl ceramide	V	△
**GD1**	Ganglioside GD1	△	△
**GD2**	Ganglioside GD2	△	
**GD2-OAc**	Ganglioside GD2 OAc	△	
**GD3**	Ganglioside GD3	△	△
**GD3-OAc**	Ganglioside GD3 OAc	△	
**GM1**	Ganglioside GM1	△	△
**GM2**	Ganglioside GM2	△	△
**GM3**	Ganglioside GM3	V	V
**GT1**	Ganglioside GT1	△	
**HexCer**	Hexosylceramide	V	V
**LPA**	Lyso-phosphatidate	V	
**LPC**	Lyso-phosphatidylcholine	V	V
**LPC O-**	Lyso-phosphatidylcholine (-ether)	V	△
**LPE**	Lyso-phosphatidylethanolamine	V	V
**LPE O-**	lyso-phosphatidylethanolamine (-ether)	V	V
**LPG**	Lyso-phosphatidylglycerol	V	
**LPI**	Lyso-phosphatidylinositol	V	△
**LPS**	Lyso-phosphatidylserine	V	△
**PA**	Phosphatidic acid	V	V
**PC**	Phosphatidylcholine	V	V
**PC O-**	Phosphatidylcholine (-ether)	V	V
**PE**	Phosphatidylethanolamine	V	V
**PE O-**	Phosphatidylethanolamine (-ether)	V	V
**PG**	Phosphatidylglycerol	V	V
**PI**	Phosphatidylinositol	V	V
**PS**	Phosphatidylserine	V	V
**SM**	Sphingomyelin	V	V
**TAG**	Triacylglycerol	V	△

△: detected but filtered out; V: detected and included in the analysis

(A)

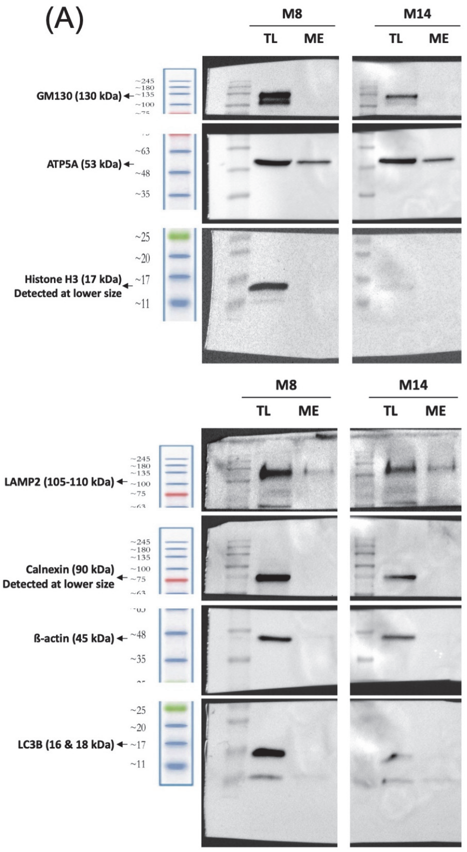

(B)

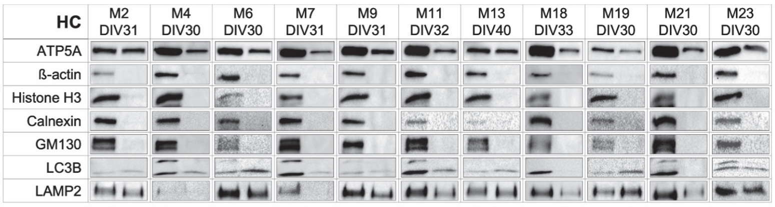

HC	M2 DIV31	M4 DIV30	M6 DIV30	M7 DIV31	M9 DIV31	M11 DIV32	M13 DIV40	M18 DIV33	M19 DIV30	M21 DIV30	M23 DIV30
ATP5A	84.91 %	37.82 %	47.71 %	24.79 %	46.93 %	33.71 %	85.39 %	24.73 %	46.57 %	25.23 %	43.02 %
ß-actin	0.04 %	2.84 %	1.71 %	0 %	0.56 %	0.03 %	1.45 %	2.03 %	8.19 %	1.24 %	1.01 %
Histone H3	3.91 %	0 %	0 %	0 %	0.21 %	0.2 %	5.71 %	0 %	0 %	0 %	0.59 %
Calnexin	3.05 %	0 %	1.22 %	0 %	0.46 %	3.31 %	16.91 %	0 %	2.69 %	1.51 %	1.82 %
GM130	6.37 %	0.42 %	1.86 %	0.14 %	0.54 %	0.16 %	2.99 %	0 %	2.25 %	0 %	5.05 %
LC3B	158.51 %	4.5 % /40.07 %	0 % /214.64 %	1.16 % /9.95 %	2.55 % /50.63 %	1.94 % /26.45 %	173.6 %	- /3.77 %	- /239.39 %	2.18 % /0.38 %	13.59 % /62.92 %
LAMP2	83.45 %	4.83 %	68.73 %	2.46 %	57.31 %	47.08 %	120.29 %	30.38 %	135.47 %	28 %	73.28 %

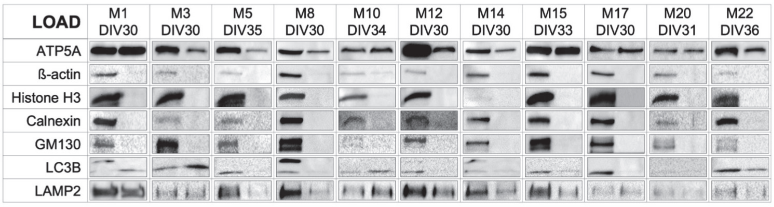

LOAD	M1 DIV30	M3 DIV30	M5 DIV35	M8 DIV30	M10 DIV34	M12 DIV30	M14 DIV30	M15 DIV33	M17 DIV30	M20 DIV31	M22 DIV36
ATP5A	109.17 %	36.09 %	23.45 %	42.84 %	116.53 %	27.53 %	39.69 %	96.29 %	102.62 %	84 %	49.82 %
ß-actin	1.98 %	5.94 %	1.5 %	4.29 %	30.73 %	0.93 %	0.04 %	0.23 %	0 %	3.67 %	1.66 %
Histone H3	0.75 %	0 %	0.81 %	0.8 %	0 %	0 %	0.77 %	0 %	0 %	0.44 %	0 %
Calnexin	1.47 %	2.32 %	0.89 %	0.62 %	0 %	1.65 %	1.35 %	0.57 %	0 %	2.85 %	1.45 %
GM130	8.94 %	0.59 %	0.21 %	0.13 %	2.57 %	4.92 %	0.24 %	2.07 %	0 %	0.2 %	0.55 %
LC3B	1.46 % /407.77 %	- /233.02 %	- /3.9 %	0.73 % /18.71 %	- /214.75 %	0.87 % /8.12 %	3.26 % /36.45 %	- /34.62 %	- /5.7 %	- /43.46 %	- /39.37 %
LAMP2	75.61 %	100.33 %	17.74 %	29.02 %	162.55 %	52.86 %	37.96 %	33.62 %	113.61 %	61.73 %	53.51 %

Supplementary Figure S1. Purity of mitochondria. Mitochondrial extracts (ME) and total cell lysates (TL) were analyzed for mitochondrial (ATP5A), cytoplasmic (β-ACTIN), nuclear (HISTONE H3), endoplasmic reticulum (CALNEXIN), Golgi (GM 130), (auto)phagosome (LC3B), and lysosome (LAMP2) markers. (**A**) Example of gels with protein standards depicting molecular weights. The microtubule-associated protein 1A/1B light chain 3B (LC3B) marker detects a larger nucleus- and cytoplasm-located form of the protein (LC3-I, 18kDa) and a smaller lipidated membrane-associated form (LC3B-II, 16kDa) which is attached to phagophores or autophagosomes [1, 2] (**B**) Images of Western blot gels for ME and TL samples used in this study. The purity of ME isolates was determined by densitometry and calculating the percent band signal of the ME from the corresponding TL fraction. Samples were coded for blind analysis. Day *in vitro* (DIV) depicts day of astrocyte differentiation on which cells were lysed, and mitochondria were extracted. HC: Healthy controls; LOAD: Late-onset Alzheimer's disease.

(A)

Cardiolipin measurements from whole cell astrocytes (before filtering by occupational threshold)

Species	C2	C4	C6	C7	C9	C11	C13	C18	C19	C21	C23	C1	C3	C5	C8	C10	C12	C14	C15	C17	C20	C22
CL 66:4;0																					23.64	
CL 68:4;0			15.51					26.88	20.90		21.76			31.71							40.15	
CL 70:4;0	14.83		13.59								19.21			33.75							34.73	
CL 70:5;0	13.10		15.61																			
CL 70:6;0													30.18					31.21				31.22
CL 72:4;0													38.62									

Cardiolipin measurements from astrocyte mitochondrial-enriched fractions (before filtering by occupational threshold)

Species	M2	M4	M6	M7	M9	M11	M13	M18	M19	M21	M23	M1	M3	M5	M8	M10	M12	M14	M15	M17	M20	M22
CL 66:4;0																						
CL 68:4;0																						
CL 70:4;0																						
CL 70:5;0																						
CL 70:6;0																						
CL 72:4;0																						

HC
LOAD

(B)

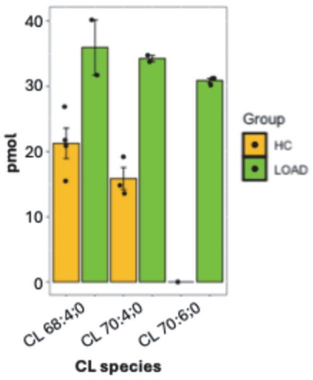

Species	ave HC	STD HC	ave LOAD	STD LOAD	% LOAD	*p* value
CL 68:4;0	21.26	4.65	35.93	5.96	169%	0.117
CL 70:4;0	15.88	2.95	34.24	0.70	216%	0.005
CL 70:6;0	-	-	30.87	0.60	-	-

(C)

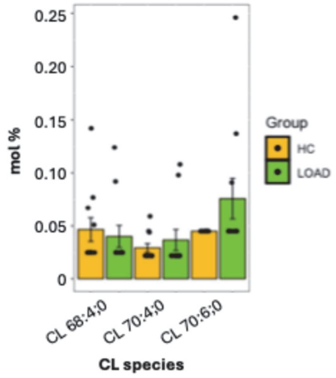

Species	ave HC	SEM HC	ave LOAD	SEM LOAD	% LOAD	*p* value
CL 68:4;0	0.047	0.011	0.040	0.010	87%	0.685
CL 70:4;0	0.030	0.004	0.037	0.010	125%	0.504
CL 70:6;0	0.045	0.000	0.076	0.019	167%	0.140

Supplementary Fig. S2. Analysis of cardiolipin (CL). (**A**) Shotgun lipidomics raw data for CL on whole cell astrocytes (upper table) or mitochondria-enriched fractions (lower table) after filtration with a signal-to-noise ratio of 5. The secondary human astrocyte control cell line was purchased from ScienCell, Carlsbad, CA (#1800). Lipid values are in pmol. Calculated amounts per protein input material were in the ranges of 0.04 – 0.43 pmol/μg protein (ave 0.20 ± 0.14 pmol/μg protein). (**B**) Filtration of unimputed CL data shown in (**A**) with an occupational threshold of 0.3, identified 3 CL species, CL 68:4;0, CL 70:4;0, and CL 70:6;0 that are increased in LOAD-associated whole cell astrocytes. CL 70:4;0 was significantly elevated (p = 0.005), calculated by two-tailed t-test. (**C**) Filtration of CL data

with our analysis criteria, i.e., mol% and imputation with half the minimum detection, and using an occupational threshold of 0.3, identified the same 3 CL species, CL 68:4;0, CL 70:4;0, and CL 70:6;0 as shown in (**B**). CL 70:4;0 and CL 70:6;0 are increased in LOAD cells but data didn't reach significance.

References

1. Baeken, M. W.; Weckmann, K.; Diefenthaler, P.; Schulte, J.; Yusifli, K.; Moosmann, B.; Behl, C.; Hajieva, P., Novel Insights into the Cellular Localization and Regulation of the Autophagosomal Proteins LC3A, LC3B and LC3C. Cells 2020, 9, (10).
2. Kabeya, Y.; Mizushima, N.; Ueno, T.; Yamamoto, A.; Kirisako, T.; Noda, T.; Kominami, E.; Ohsumi, Y.; Yoshimori, T., LC3, a mammalian homologue of yeast Apg8p, is localized in autophagosome membranes after processing. EMBO J 2000, 19, (21), 5720-8.
